# Assessment of Coproduction of Ethanol and Methane from *Pennisetum purpureum*: Effects of Pretreatment, Process Performance, and Mass Balance

**DOI:** 10.1021/acssuschemeng.1c02010

**Published:** 2021-08-05

**Authors:** Peiwen Wu, Xihui Kang, Wen Wang, Gaixiu Yang, Linsong He, Yafeng Fan, Xingyu Cheng, Yongming Sun, Lianhua Li

**Affiliations:** † Guangzhou Institute of Energy Conversion, Chinese Academy of Sciences, No. 2, Nengyuan Road, Guangzhou 510640, China; ‡ Key Laboratory of Ministry of Education for Water Quality Security and Protection in Pearl River Delta, Guangdong Provincial Key Laboratory of Radionuclides Pollution Control and Resources, School of Environmental Science and Engineering, Guangzhou University, No. 230, Wai Huan Xi Road, Guangzhou 510006, China; § Guangzhou Institute of Energy Conversion, CAS Key Laboratory of Renewable Energy, Chinese Academy of Sciences, No. 2, Nengyuan Road, Guangzhou 510640, P.R. China; ∥ Guangdong Key Laboratory of New and Renewable Energy Research and Development, No. 2, Nengyuan Road, Guangzhou 510640, P.R. China; ⊥ MaREI Centre, Environmental Research Institute, University College Cork, 4 Lee Road, Sunday’s Well, Cork, Ireland

**Keywords:** Energy crops, Pretreatment, Anaerobic digestion, Biofuels, Material flow analysis

## Abstract

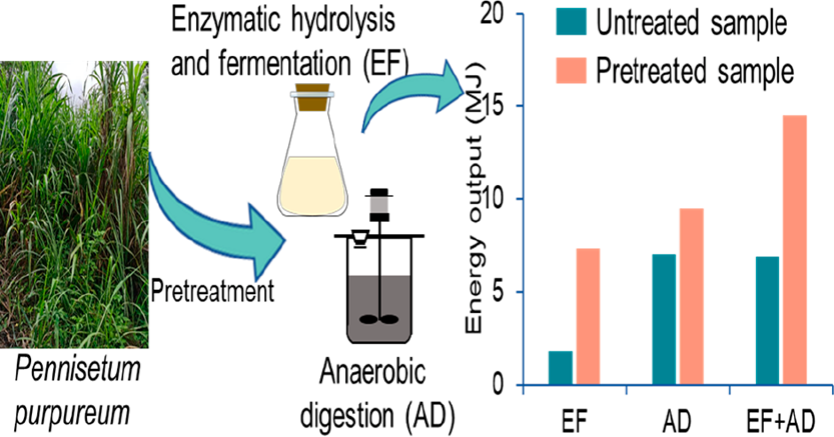

To overcome the structural complexity and improve the bioconversion efficiency of *Pennisetum purpureum* into bioethanol or/and biomethane, the effects of ensiling pretreatment, NaOH pretreatment, and their combination on digestion performance and mass flow were comparatively investigated. The coproduction of bioethanol and biomethane showed that 65.2 g of ethanol and 102.6 g of methane could be obtained from 1 kg of untreated *Pennisetum purpureum*, and pretreatment had significant impacts on the production; however, there is no significant difference between the results of NaOH pretreatment and ensiling-NaOH pretreatment in terms of production improvement. Among them, 1 kg of ensiling-NaOH treated *Pennisetum purpureum* could yield 269.4 g of ethanol and 144.5 g of methane, with a respective increase of 313.2% and 40.8% compared to that from the untreated sample; this corresponded to the final energy production of 14.5 MJ, with the energy conversion efficiency of 46.8%. In addition, for the ensiling-NaOH treated *Pennisetum purpureum*, the energy recovery from coproduction (process III) was 98.9% higher than that from enzymatic hydrolysis and fermentation only (process I) and 53.6% higher than that from anaerobic digestion only (process II). This indicated that coproduction of bioethanol and biomethane from *Pennisetum purpureum* after ensiling and NaOH pretreatment is an effective method to improve its conversion efficiency and energy output.

## Abstract 2

We investigated the effects of pretreatment on the coproduction (ethanol and methane) performance and material flow from *Pennisetum purpureum*.

## Introduction

Bioenergy and biofuels production originating from biomass has drawn increasing attention due to its advantages of protecting the environment and relieving the urgent demand for fossil energy.^1^ Lignocellulosic biomass, including hardwood, softwood, and herbaceous plants, with an annual yield of up to 200 billion tons globally, offers an inexpensive and abundant resource for biofuels production.^2,3^ However, the inherent complex structure of lignocellulosic biomass, including high cellulose crystallinity, carbohydrates-lignin complexity, and high lignin content, resists the attack on carbohydrates by microorganisms or enzymes during biological conversion, resulting in low conversion efficiency and thus a low biofuel production.^4,5^ In order to promote the bioconversion efficiency, pretreatment prior to the biological conversion process is considered to efficiently destroy the complex structure, and various pretreatment methods have been developed.^6^

Pretreatment methods are usually classified into physical, chemical, and biological categories and their combination.^7,8^ Among them, physical pretreatment includes mechanical pulverization, ultrasound, and radiation; chemical pretreatment includes acid/alkaline pretreatment,^9,10^ oxidation pretreatment, and ionic liquid pretreatment;^11^ while biological pretreatment mainly consists of enzyme pretreatment and microbial/fungi pretreatment.^12^ Each has its own merits and drawbacks. Among biological pretreatment methods, ensiling pretreatment has received wide research interests due to its advantages in low external energy demand, preserving the nutrients of raw material, and providing a long-term stable supply of material.^13^ The main effect of ensiling pretreatment is that hemicellulose is hydrolyzed to monosaccharides such as glucose and xylose, which are then converted to short-chain organic acids such as lactate and acetate through anaerobic microbial fermentation; as such the recalcitrance of lignin-carbohydrate complexity is reduced, increasing the accessibility of cellulose to microorganisms and enzymes.^14,15^ Moreover, intermediates produced from ensiling pretreatment such as lactic acid can be easily converted into gaseous biofuel (such as biogas). Zhao et al. reported that, compared with raw switchgrass, biomethane production from the anaerobic digestion (AD) of ensiling-treated switchgrass was improved by 33.6%.^16^ It was also reported that ensiling pretreatment increased the ethanol yield from sugar beet pulp by nearly 50%.^17^ Except for ensiling pretreatment, other biological pretreatments have also been proven to improve the fermentation performance of biomass. For example, the biogas production from microalgae increased by more than 21% after enzymatic pretreatment;^18^ Wyman et al. reported that the biogas production from corn stover pretreated with white-rot fungi increased by 19%.^19^ Chemical pretreatment methods refer to the use of chemicals (such as alkaline, acids, and ionic liquid) during the process, which have been widely accepted to increase the methane yield in the AD process;^20^ among these methods, alkaline pretreatment, with NaOH as the most employed and effective chemical reagent,^21^ has drawn extensive attention with following advantages: (1) it can be carried out under mild conditions (atmospheric pressure under 100 °C);^3,7^ (2) the hydroxide ion (OH^–^) can remove most lignin by breaking the ester bonds between xylan and lignin, and partial hemicellulose by weakening the hydrogen bond between cellulose and hemicellulose,^22^ which facilitates the accessibility of cellulose to microorganisms and enzymes. Kang et al. investigated the effect of NaOH pretreatment on the digestion performance of *Pennisetum Hybrid*, reporting that the methane yield increased from 249.3 mL/g volatile solid (VS) to 301.7 mL/g VS under the condition of 35 °C for 24 h.^23^ Kataria et al. reported that the ethanol yield from NaOH treated Kans grass under the condition of 120 °C for 120 min increased to 0.38 g/g substrate.^24^ To the best of our knowledge, although both ensiling and NaOH pretreatment are popular methods employed for improving bioenergy and biofuel production from raw lignocellulosic material, the effect of integrated ensiling-NaOH pretreatment on biofuel production has not been studied yet. Integrated ensiling pretreatment and NaOH pretreatment can not only ensure the long-term supply of raw materials but also remove lignin and improve biofuel production efficiency.

*Pennisetum purpureum*, a kind of herbaceous plants, has characteristics of high biomass production, strong adaptability to the environment, and high cellulose content, making it a potential candidate for biofuel production.^25^ Previous studies have proved that *Pennisetum purpureum* is a promising feedstock for ethanol production, with a yield of 4.3 mg/mL.^26^ Stillage, the residues after ethanol fermentation, contains high nondegraded carbohydrates content and other organic matters, which has a high chemical oxygen demand (COD) load and is considered to have a considerable pollution potential, especially for industrial-scale bioethanol plant;^27^ it is reported that the amount of stillage produced is about 10 times that of bioethanol production.^28^ Thus, treating stillage with the well-established AD technique can not only reduce pollution but also produce renewable biogas, so as to make full advantage of pentose and other organic matters that cannot be utilized in ethanol fermentation.^29^ Kaparaju et al. found that the methane yield of 324 mL/g VS was obtained when the wheat straw stillage was used as raw material with a concentration of 12.8 g VS/L.^30^ Alkan-Ozkaynak et al. used corn stillage as raw material for AD and found that the biogas yield was 763 mL/g VS. These suggest that stillage is a potential substrate for AD.^31^ Moreover, Liu et al. found that the energy conversion efficiency of sugarcane bagasse after sequential bioethanol and biogas production increased up to 59.5%; this was only 35.7% for ethanol production and 23.8% for biogas production, respectively.^32^ Several studies comparing the energy output of pretreated feedstocks in ethanol production alone, methane production alone, and ethanol–methane coproduction are presented in Table 1; it can be concluded that the energy produced by the coproduction process is significantly higher than that of the single production process. However, the energy output of feedstocks after ensiling pretreatment or NaOH pretreatment in these three production processes is yet analyzed in detail. Herein, it is plausible to speculate that the coproduction of ethanol and biogas/biomethane from ensiling, NaOH and combined pretreated *Pennisetum purpureum* would greatly improve its conversion efficiency; as such it would make this feedstock more feasible for biofuel production on an industrial scale, which necessitates further study to verify this hypothesis. In addition, the introduction of material flow analysis (MFA) into a biofuel production process could provide some useful and insightful information regarding the material utilization efficiency and digestion efficiency, making it possible to concisely control the process.^33^ Previous studies focused on the mass and energy flow of ethanol–methane coproduction, however, with the carbon and nitrogen flow yet not investigated.

**Table 1:** Comparison of Energy Output from Single Production and Coproduction of Different Raw Materials and Pretreatments

raw material	pretreatment	energy output	ref
wheat straw	hydrothermal	3.6 MJ/kg TS (ethanol)	(34)
*Pennisetum purpureum*	steam explosion	3.6 MJ/kg TS (ethanol)	(35)
oat straw	steam explosion	7.4 MJ/kg TS (methane)	(36)
duckweed	/	6.8 MJ/kg TS (methane)	(37)
wheat straw	hydrothermal	9.1 MJ/kg TS (ethanol + methane)	(34)
wheat straw	combined biological and steam explosion	10.9 MJ/kg VS (ethanol + methane)	(38)

Therefore, the objectives of this study were to (1) investigate the effects of ensiling pretreatment, NaOH pretreatment, and combined pretreatment on the physicochemical structure and ethanol-methane coproduction conversion efficiency of *Pennisetum purpureum*; (2) analyze and compare the material and energy flow of *Pennisetum purpureum* under different processes, and (3) calculate and compare the energy conversion efficiency of *Pennisetum purpureum* to optimize the feasible pretreatment and biological conversion process.

## Materials and Methods

### Raw Material

*Pennisetum purpureum*, were harvested from Zengcheng district, Guangzhou City (China), in September 2019, with a plant height of 2–3 m. The harvested samples were chopped to 1–2 cm by hay cutter and smashed with a pulverizer, and then a part of the samples was frozen at −20 °C before use. The other part was vacuumed and sealed in plastic silo bags and ensiled at ambient temperature for 90 days. The composition of all samples is listed in Table 2.

**Table 2:** Physicochemical Characteristics of *Pennisetum purpureum* before and after Ensiling

parameter (unit)	untreated	ensiling
TS (%)^a^	20.4 ± 1.5	16.3 ± 0.2
VS (%)	95.1 ± 0.2	92.4 ± 1.7
C (%)	44.7 ± 0.1	43.9 ± 0.1
N (%)	0.7 ± 0.0	0.8 ± 0.0
H (%)	6.3 ± 0.0	6.1 ± 0.0
C/N	67.8 ± 1.4	57.4 ± 2.5
glucan (%)	36.9 ± 0.7	37.8 ± 0.1
xylan (%)	18.4 ± 0.0	19.0 ± 0.6
lignin (%)	10.9 ± 0.5	14.1 ± 0.1

a Total solid (TS) is calculated based on wet weight; others are based on the dry weight.

NaOH was purchased from Macklin Bio-Chem Technology Co. Ltd. The commercial cellulase with an activity of 144 FPU/g powder was purchased from Imperial Jade Biotechnology Co. Ltd. (Ningxia, China), which was extracted from the fermentation broth of *Penicillium sp.. Saccharomyces cerevisiae* Y2034 used for ethanol production was obtained from the National Center for Agricultural Utilization Research (U.S.A.),^39^ which can only utilize hexose with an ethanol fermentation efficiency of 85–90%.

### Experimental Design and Process

**Figure 1:**
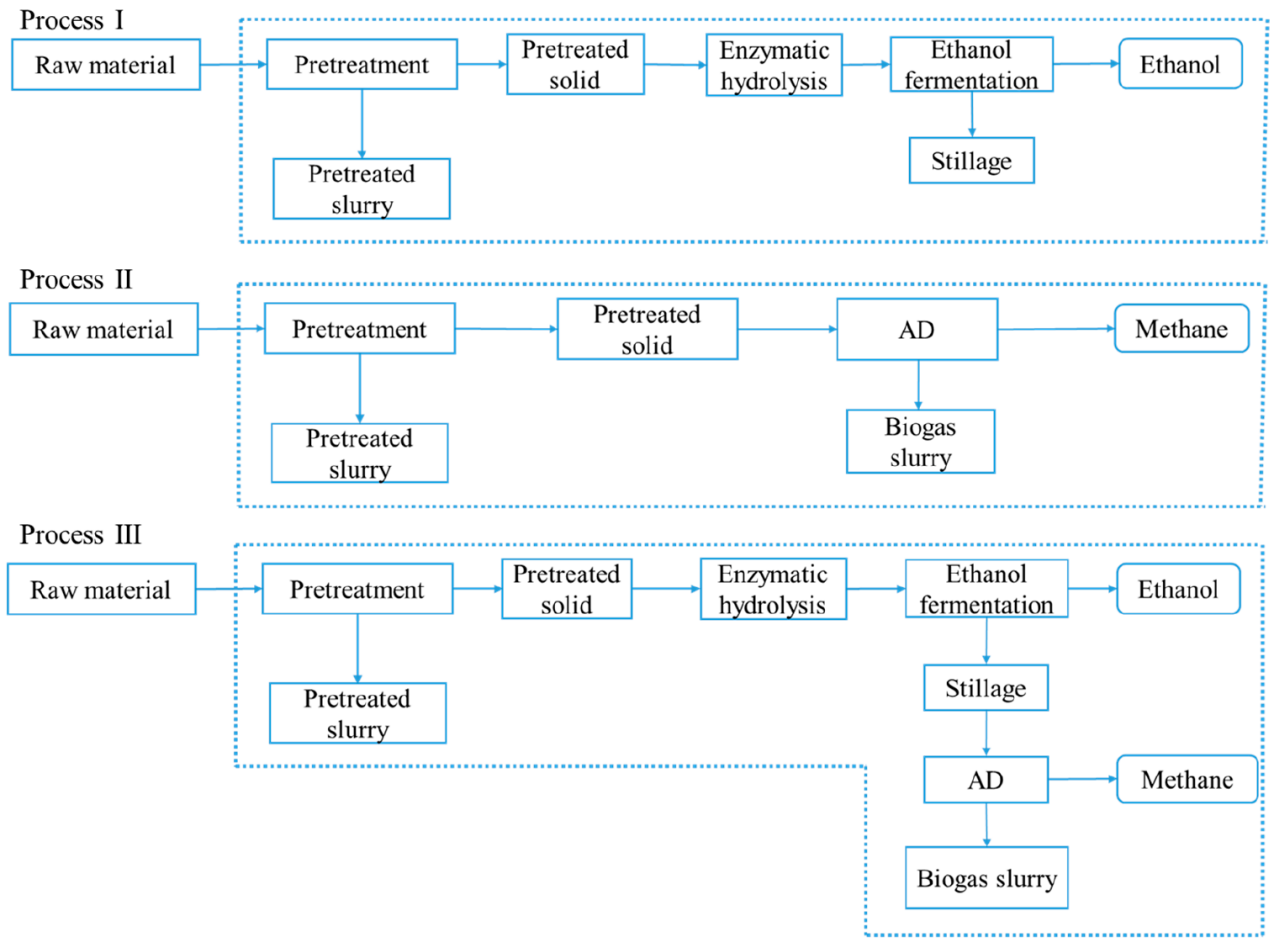
Simplified flowchart for three processes (AD: anaerobic digestion).

To compare the effects of ensiling pretreatment, NaOH pretreatment, and their combination on the bioconversion efficiency of *Pennisetum purpureum*, three processes were designed in this study, which was illustrated in Figure 1. Process I was performed to produce ethanol via enzymatic hydrolysis and fermentation, and process II was performed to produce biogas through AD from untreated and pretreated *Pennisetum purpureum*, respectively. Process III integrated AD of stillage (including both solid and liquid fractions) from Process I after ethanol fermentation to achieve coproduction of ethanol and biogas.

### Pretreatment

Ensiling pretreatment was conducted in a vacuumed and sealed plastic silo bag, and then stored at ambient temperature for 90 days.

According to a previous study where the ethanol production from NaOH pretreated sugarcane bagasse increased to nearly 20 g/L, the alkaline pretreatment condition was chosen to be 2% NaOH (w/v), a solid-to-liquid ratio of 1:20 (w/v) based on total solids (TS), at 80 °C for 2 h with 150 rpm.^40^ Sequential ensiling and NaOH pretreatment were set up under the same condition, with the ensiled samples underwent the NaOH pretreatment. After pretreatment, the samples were centrifuged for solid–liquid separation. The solid fraction was washed with distilled water to a neutral pH and then stored at −20 °C for the enzymatic hydrolysis and fermentation. The liquid fraction was stored to determine the concentration of total carbon (TC) and total nitrogen (TN).

### Enzymatic Hydrolysis and Ethanol Fermentation

Before fermentation, enzymatic hydrolysis of the pretreated samples was performed to achieve the conversion of polysaccharides into monosaccharides. The sterilized pretreated samples were added into the aseptic 0.05 M acetate buffer (pH 4.8) at a solid concentration of 10%; the buffer contains 5 g/L yeast extract, 5 g/L peptone, 5 g/L KH_2_PO_4_, 0.2 g/L (NH_4_)_2_SO_4_, and 0.4 g/L MgSO_4_·7H_2_O. The enzymatic hydrolysis experiment was carried out at the cellulase loading of 20 FPU/g cellulose of substrate and shaken at the condition of 50 °C and 150 rpm for 72 h. The hydrolysates (including both solid and liquid fractions) after enzymatic hydrolysis were inoculated with the activated yeast strain which was cultivated at 30 °C and 150 rpm for 16 h in a medium containing 20 g/L peptone, 10 g/L yeast extract, and 20 g/L glucose; the reaction system was then placed in a shaker incubator at 30 °C and 150 rpm for 72 h. For high-performance liquid chromatography (HPLC) analysis, samples were taken at intervals of 12 h during enzymatic hydrolysis and 24 h during ethanol fermentation.

All fermentation slurries were distilled with a rotary vacuum evaporator at 70 °C and −0.1 MPa for 7 min to separate the ethanol from the fermented liquid.

### Anaerobic Digestion

The anaerobic digestion experiments of untreated or pretreated samples (process II) and stillage (process III) were performed at an automatic methane potential test system (Bioprocess Control Sweden AB, AMPTS II).^41^ The untreated or pretreated samples that performed anaerobic digestion directly were denoted AD. For example, AD of samples after ensiling pretreatment, NaOH pretreatment, and ensiling-NaOH pretreatment are coded AD-ensiling, AD-NaOH, and AD-ensiling-NaOH, respectively. While the stillage (including both solid and liquid fractions) from the process I performed AD was denoted ensiling, NaOH, and ensiling-NaOH after ensiling, NaOH, and ensiling-NaOH pretreatment. The inocula were added according to the VS content of the solid part after distillation the untreated/pretreated sample, and the ratio of samples to inoculum was 1:2 based on VS. The inocula-only (400 mL) reactors were used as controls. The actual methane yield was calculated by eq 1. All reactors were flushed with nitrogen gas for 5 min to guarantee an anaerobic condition. The temperature was controlled at 37 ± 1 °C. Each condition was set in triplicate. The entire period of this experiment was 30 days.
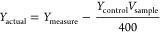
1where *Y*_actual_ is the specific methane yield (mL/g VS) from samples, *Y*_sample_ is the sample measured methane yield (mL/g VS), *Y*_control_ is the methane production of the inocula-only (mL/g VS), *V*_sample_ is the volume of inocula added in the sample reactor (mL), and 400 is the volume of inocula added in the control reactor. The inocula for biogas production were granule sludge which were cultivated with cellulose and peptone in the laboratory before use; the pH value, TS content and VS content of the inocula were 7.5, 1.7 ± 0.1%, and 1.5 ± 0.0%, respectively.

### Analytical Methods

The content of TS, VS, C, and N in the solid fraction from different processes was determined according to the previously described method.^42^ The samples were dried at 105 °C to a constant weight and then placed in a muffle furnace and incinerated at 550 °C for 2 h to calculate TS and VS. C and N content in the solid fraction were analyzed by Vario EL cube (elementar, Germany). A calorimeter (IKA C2000, Germany) was used to measure the calorific value (CV) of the samples.^43^ The TC and TN concentrations of the liquid fraction after NaOH pretreatment, ethanol fermentation, and AD were analyzed by Vario TOC (elementar, Germany) at 850 °C with an oxygen flow rate of 230 mL/min. The carbohydrates (glucan and xylan) and lignin contents of all samples were analyzed in accordance with the description of National Renewable Energy Laboratory (NREL).^44^ The monomeric sugars and ethanol concentration were tested by the HPLC system (Waters e2698, U.S.A.) equipped with SH1011 (Shodex) at 50 °C with 5 mM H_2_SO_4_ as the mobile phase at a flow rate of 0.5 mL/min. The surface morphologies of untreated and pretreated samples were imaged by scanning electron microscopy (SEM, Hitachi S4800) at an accelerating voltage of 2.0 kV.^45^

### Analysis of Mass and Energy Flow

The flow of mass, element (C and N), and energy was systematically evaluated by MFA according to the study by Brunner and Rechberger.^46^ A software for substance flow analysis (STAN 2.6.801) was used to establish the MFA system model, and the data were processed and optimized by IAL-IMPL2013 algorithm to achieve material balance. The results of MFA were presented by graphics according to the study by Niu et al.^43^

### Data Calculation and Statistical Methods

Glucan recovery and lignin removal were calculated as per eqs 2 and 3, respectively, as follows:
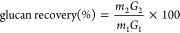
2
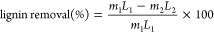
3where *m*_1_ and *m*_2_ are the mass of the sample before and after pretreatment (g), *G*_1_ and *G*_2_ are the percentages of glucan in the sample before and after pretreatment (%), and *L*_1_ and *L*_2_ are the percentages of lignin in the sample before and after pretreatment (%).

Enzymatic hydrolysis yield and ethanol yield were respectively calculated as follows:
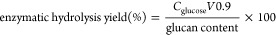
4
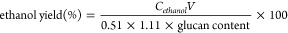
5where *C*_glucose_ is the concentration of glucose during enzymatic hydrolysis (mg/mL), *C*_ethanol_ is the concentration of ethanol during fermentation (mg/mL), glucan content is the mass of glucan in samples (mg), *V* is the volume of fermentation liquid (mL), 0.9 is the dehydration coefficient of glucose converted to glucan, 0.51 is the theoretical coefficient for converting glucose into ethanol, and 1.11 is the theoretical coefficient of conversion of glucan to glucose.^47^

A software of SPSS 19.0 was applied to analyze the statistical difference of the data, and a one-way analysis of variance was used. The variance level was 0.05%.

## Results and Discussion

### Enzymatic Hydrolysis and Fermentation of *Pennisetum purpureum* under Process I

**Figure 2:**
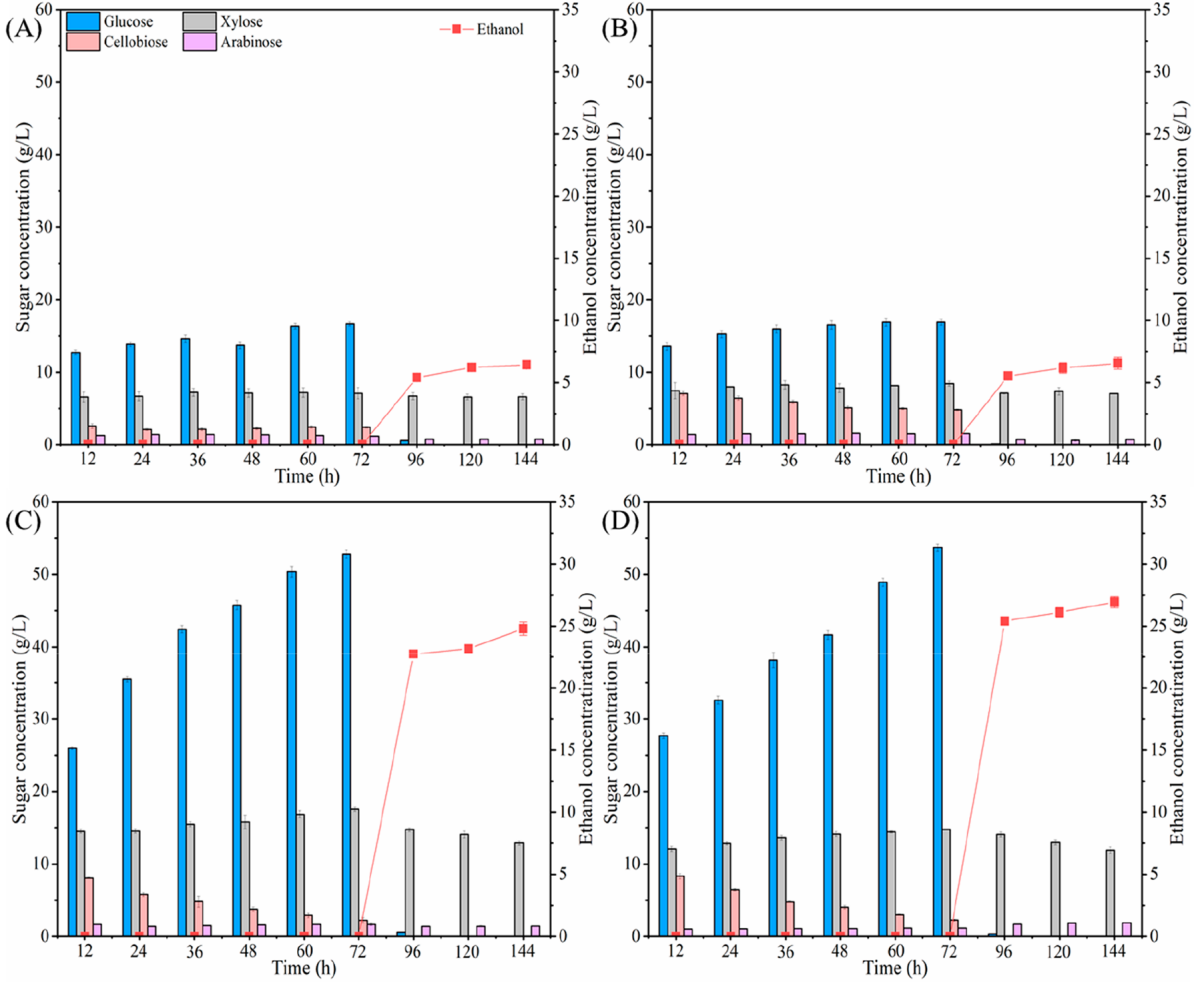
Monomeric sugars and ethanol concentration of untreated and pretreated *Pennisetum purpureum*. A: untreated sample; B: ensiling treated sample; C: NaOH treated sample; D: ensiling-NaOH treated sample.

The concentration of monomeric sugars and ethanol during the respective enzymatic hydrolysis and fermentation process (process I) are shown in Figure 2. During the enzymatic hydrolysis, it was observed that the concentrations of glucose, xylose, cellobiose, and arabinose varied for all samples. With the time prolonged, the concentration of glucose increased significantly, while the xylose concentration was almost unchanged. For the untreated sample, the glucose concentration yielded at 16.7 g/L after 72 h enzymatic hydrolysis (Figure 2A). For ensiling pretreated sample, the glucose concentration was 16.9 g/L at the end of enzymatic hydrolysis (Figure 2B), with an insignificant increase of 1.2% when compared with that from the untreated sample (*p* > 0.05). For the NaOH pretreated sample, the glucose concentration was 52.8 g/L (Figure 2C), which increased by 216.7% compared to that from the untreated sample (*p* < 0.05). For the ensiling-NaOH pretreated sample, the glucose concentration was 53.7 g/L after 72 h enzymatic hydrolysis (Figure 2D), resulting in an increase of 222.0% compared to that from the untreated sample (*p* < 0.05), while the xylose and arabinose concentrations were almost constant throughout the enzymatic hydrolysis.

After the enzymatic hydrolysis experiments (72 h), the hydrolysates (including solid and liquid fraction) were added into a fermentation reactor with activated yeast strain for ethanol fermentation. As also shown in Figure 2, the ethanol concentration dramatically increased after 24 h incubation. After 24 h incubation, the ethanol concentration was 5.4 g/L for the untreated sample (Figure 2A), 5.5 g/L for the ensiling treated sample (Figure 2B), 22.8 g/L for the NaOH treated sample (Figure 2C), and 25.4 g/L for the ensiling-NaOH treated sample (Figure 2D); these accounted for 84.2–94.4% of the total final ethanol concentration, which was 6.4 g/L for the untreated sample, 6.6 g/L for the ensiling treated sample, 24.8 g/L for the NaOH treated sample, and 26.9 g/L for the ensiling-NaOH treated sample, respectively. Meanwhile, the glucose concentration sharply decreased to 0.12–0.62 g/L, while the xylose concentration remained unchanged. These results agreed with Wang et al.,^48^ who reported that the yeast strain can timely ferment glucose into ethanol but cannot assimilate xylose. In addition, the ethanol concentration from the ensiling-NaOH treated sample was increased by 318.8% compared to that from the untreated sample, 310.9% compared to that from the ensiling treated sample, and 8.6% compared to that from the NaOH treated sample (*p* < 0.05). It was worth mentioning that the ethanol yield from the NaOH treated sample and the ensiling-NaOH treated sample was three times that from the ensiling treated sample, indicating that NaOH pretreatment and sequential ensiling and NaOH pretreatment of *Pennisetum purpureum* both are effective pretreatment methods to increase the ethanol yield. Meanwhile, it also suggested that ensiling-NaOH pretreatment is a feasible method that can not only realize the continuous supply of raw material but also improve the fermentation performance. This can be attributed to the removal of lignin after NaOH pretreatment, as the lignin removal was 57.2% for ensiling treated samples, and the relative glucan content was up to 62.1% (Table 3). In addition, the changes in the surface structure after pretreatment can also explain the increased efficiency of enzymatic hydrolysis and ethanol yield. The untreated and ensiling treated sample exhibited a smooth and orderly surface structure, but after NaOH pretreatment, the surface became rough and shaggy (Supporting Information), which increases the accessibility of cellulose. The ethanol concentration produced in this study are comparable with elephant grass and bagasse, with a respective yields of 26.1^25^ and 15.0 g/L.^49^

**Table 3:** Compositions of Untreated and Pretreated *Pennisetum purpureum*^a^

feedstock	pretreatment	glucan (%)	xylan (%)	lignin (%)	other (%)
*Pennisetum purpureum*	untreated	36.9 ± 0.7c	18.4 ± 0.0c	10.9 ± 0.5b,c	33.8 ± 0.2a
	ensiling	37.8 ± 0.1c	19.0 ± 0.6c	14.1 ± 0.1a	29.2 ± 0.6b
	NaOH	59.5 ± 0.8b	24.5 ± 0.3a,b	8.7 ± 0.0d	7.3 ± 0.5c
	ensiling-NaOH	62.1 ± 0.4a	24.2 ± 0.0a,b	10.4 ± 0.4b,c	3.3 ± 0.0d

a Letters indicate that the values in the same column are significantly different (*p* < 0.05).

### Anaerobic Digestion Performance of *Pennisetum purpureum* under Process II

**Figure 3:**
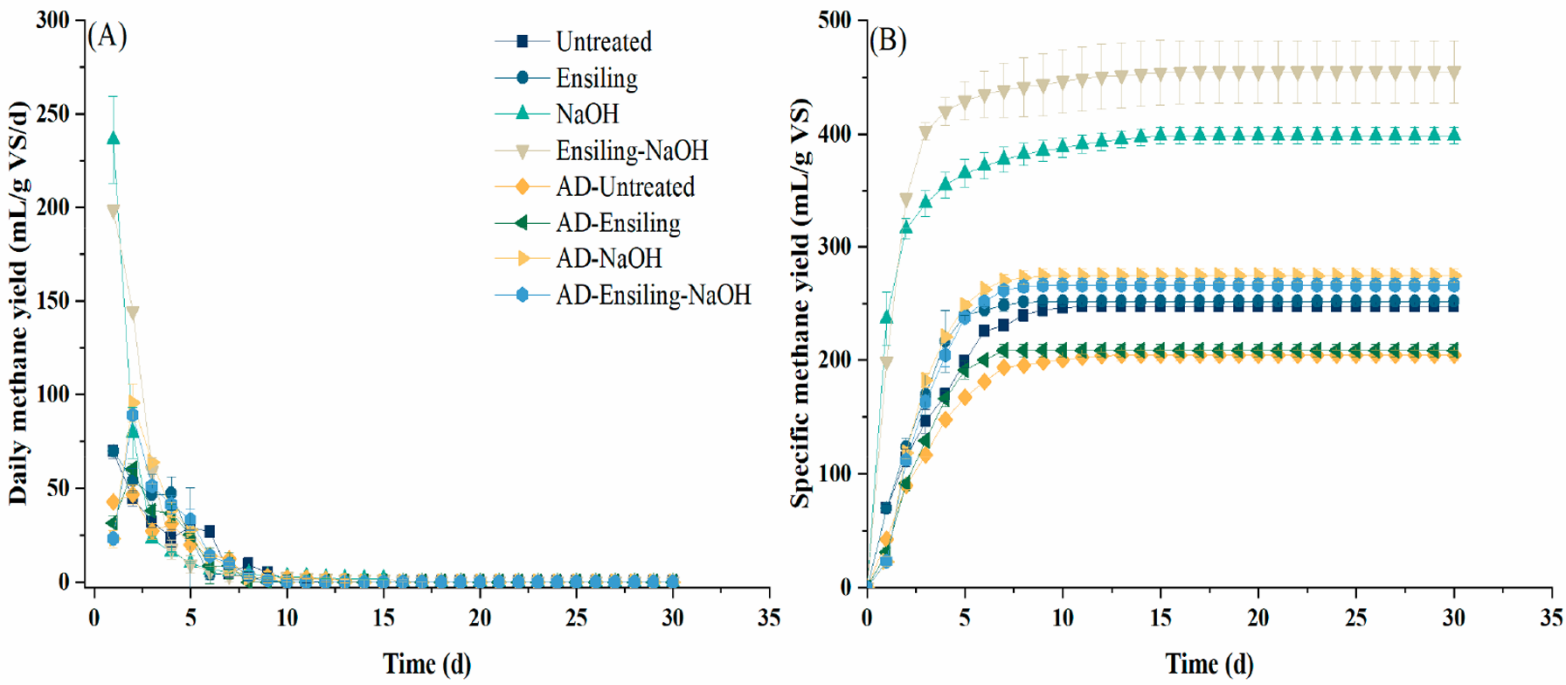
Daily methane yield (A) and specific methane yield (B) of samples.

The daily and cumulative methane yield of untreated and pretreated *Pennisetum purpureum* (process II) are shown in Figure 3. For untreated samples (AD-untreated), the highest daily methane yield was 46.8 mL/g VS/d (Figure 3A) on day 2; this increased to 60.1 mL/g VS/d after ensiling pretreatment (AD-ensiling). For NaOH treated samples (AD-NaOH), the daily methane yield peaked at 95.4 mL/g VS/d on day 2, with an increase of 104.1% compared to that of untreated samples (*p* < 0.05). After ensiling-NaOH pretreatment (AD-ensiling-NaOH), the highest daily methane yield was 89.0 mL/g VS/d on day 2, which was increased by 90.3%, when compared to that from the untreated sample (*p* < 0.05). As shown in Table 4 and Figure 3B, the specific methane yield of the untreated sample was 204.6 mL/g VS; this increased to 208.9 mL/g VS after ensiling pretreatment (*p* > 0.05). After NaOH pretreatment, the specific methane yield was 274.5 mL/g VS, corresponding to an increase of 34.1% compared to that of the untreated sample (*p* < 0.05). The specific methane yield of ensiling-NaOH treated samples was 266.1 mL/g VS, which increased by 30.1% when compared with that of the untreated sample (*p* < 0.05). These results agreed with Costa et al.,^50^ who investigated the effect of NaOH pretreatment on the digestion performance of sugar cane bagasse and reported that the methane production from NaOH treated samples increased to 313.4 mL/g dry substrates.

**Table 4:** Specific Methane Yield of Samples under Processes II and III^a^

porduction process	pretreatment	specific methane yield (mL/g VS)
process II	untreated	204.6 ± 4.9g
	ensiling	208.9 ± 5.4g
	NaOH	274.5 ± 5.9c,d,e
	ensiling-NaOH	266.1 ± 5.8c,d,e,f
process III	untreated	246.9 ± 1.8d,e,f
	Ensiling	251.7 ± 4.3c,d,e,f
	NaOH	398.6 ± 7.0b
	ensiling-NaOH	454.8 ± 27.7a

a Letters a–g indicated that the values in the same column are significantly different (*p* < 0.05).

It was noticed that the specific methane from the NaOH treated sample was significantly higher than that from the untreated and ensiling treated sample (*p* < 0.01). This might be explained by the different lignin content in untreated and pretreated samples (Table 3). Herrmann et al. reported that higher lignin content has a negative impact on specific methane yield from lignocellulosic biomass such as Miscanthus and ryegrass, due to its cross-linkage with homocellulose, which reduces the biodegradability of homocellulose.^51^ Similar results have been reported by Sambusiti et al.,^52^ who found that the specific methane yield of NaOH treated sorghum increased by 18.80% with a lignin reduction of 63%. Under the conditions of 4 g NaOH/100 g TS and 55 °C for 24 h, the methane yield of sunflower stalk varieties increased by 29–44%, and the lignin removal was 23.3–36.3%.^53^ Kang et al. also found that the lignin removal was 58.9% when the *Pennisetum Hybrid* was pretreated with 2% NaOH at 35 °C for 24 h, and the methane yield was increased by 21.0% under this condition.^23^

### Anaerobic Digestion Performance of Stillage Samples of *Pennisetum purpureum* under Process III

The daily and cumulative methane yield from the AD of stillage after ethanol fermentation (process III) are also shown in Figure 3. For untreated samples (untreated), the highest daily methane yield was 69.5 mL/g VS/d (Figure 3A) on day 1; this was 69.9 mL/g VS/d after ensiling pretreatment (ensiling). For NaOH treated samples (NaOH), the daily methane yield peaked at 236.5 mL/g VS/d on day 1, with an increase of 240.2% compared to that from the untreated sample. After ensiling-NaOH pretreatment (ensiling-NaOH), the highest daily methane yield was 198.8 mL/g VS/d on day 1, which increased by 186.1% when compared to that from the untreated sample. As shown in Table 4 and Figure 3B, the cumulative methane yield of the untreated sample was 246.9 mL/g VS; this increased to 251.7 mL/g VS after ensiling pretreatment (*p* > 0.05). After NaOH pretreatment, the specific methane yield was 398.6 mL/g VS, corresponding to an increase of 61.4% compared to that of the untreated sample (*p* < 0.05). The specific methane yield of the ensiling-NaOH treated sample was 454.8 mL/g VS, which increased by 84.2% when compared with that of the untreated sample (*p* < 0.05). In addition, compared with the corresponding sample directly perform AD under process II, the methane yield of stillage samples under process III was respectively increased by 20.7% for the untreated sample, 20.5% for the ensiling treated sample, 45.2% for the NaOH treated sample, and 70.9% for the ensiling-NaOH treated sample. These results were similar to the literature in which the methane yield of stillage was 13.4–34.0% higher than that of pretreated barley straw.^54^ The enhanced maximum daily methane yield and cumulative methane yield could be explained by that the stillage contains more soluble pentose such as xylose and other degradable compounds, which can be easily converted to methane during the AD process.^55^ A previous study reported that utilization of xylose for biogas production could outpace its use for ethanol in terms of energy recovery; as such the AD of stillage achieved the highest energy recovery from acetic acid-pretreated corn stover after ethanol fermentation.^56^ Therefore, to better understand the mass balance and energy conversion efficiency of the substrate under different processes, it necessitates the comparisons in terms of mass flow including C and N content and energy recovery in this study.

### Material Flow and Energy Output of Three Processes

#### Variation in the Characteristics of Samples during the Process

The variation in the contents of TS, VS, C, N, TC, TN, and CV of *Pennisetum purpureum* during the coproduction process is shown in Table 5. Each step during the coproduction process has been analyzed. For the solid part of *Pennisetum purpureum*, the carbon contents were 44.7% for the untreated sample and 43.9% for ensiling treated sample, and after NaOH pretreatment, the values respectively decreased to 42.7% and 42.9% (*p* < 0.05). For the untreated sample, the carbon content in the solid part decreased to 41.0% after enzymatic hydrolysis and ethanol fermentation and further decreased to 38.2% after AD. For the ensiling treated sample, the carbon content in the solid part decreased to 40.5% after enzymatic hydrolysis and ethanol fermentation and further decreased to 38.6% after AD (*p* < 0.05). The carbon content in the solid part decreased to 39.5% for NaOH treated sample and 37.9% for ensiling-NaOH treated sample after enzymatic hydrolysis and ethanol fermentation, and further decreased to 37.6% and 35.4% after AD (*p* < 0.05), respectively. The nitrogen content in the solid part of *Pennisetum purpureum* was 0.7% for the untreated sample and 0.8% for ensiling treated sample and completely removed after NaOH pretreatment (*p* < 0.05), indicating that the NaOH pretreatment used in this study had an obvious effect on nitrogen removal. A significant increase of nitrogen content in the solid part was observed after enzymatic hydrolysis and ethanol production (*p* < 0.05); the nitrogen content increased to 2.1% for the untreated sample and 2.0% for the ensiling treated sample. Similarly, the values also increased to 2.3% for NaOH treated sample and 2.2% for ensiling-NaOH treated sample, respectively; this could be attributed to (1) the addition of cellulase, inoculum, and seed medium and (2) the protein was dissolved under alkaline condition and then precipitated in enzymatic hydrolysis and ethanol fermentation process (acidic condition). After AD, the nitrogen content in the solid part decreased to 2.0% for the untreated sample, 2.0% for the ensiling treated sample, 2.2% for the NaOH treated sample, and 2.1% for the ensiling-NaOH treated sample, respectively. The decreased nitrogen content could be attributed to the growth of methanogens during AD process which needs nitrogen, and consequently, the protein was degraded into free ammonia remaining in the liquid part.^57,58^

**Table 5:** Characteristics of *Pennisetum purpureum* at Different Stages^a^

	TS (%)	VS (%TS)	C (%)	N (%)	CV (J/g)	TC (mg/L)	TN (mg/L)
	untreated and treated sample					liquid part after pretreated	
untreated	20.4 ± 1.5a,b	95.1 ± 0.2c	44.7 ± 0.1a	0.7 ± 0.0b	16755.5 ± 96.9c		
ensiling	16.3 ± 0.2c	91.5 ± 0.7c	43.9 ± 0.1b	0.8 ± 0.0a	18007.0 ± 24.0a		
NaOH	13.9 ± 0.2c	98.9 ± 0.1a,b	42.7 ± 0.1c	0.0c	16830.0 ± 2.8c	7240.0 ± 11.1a	404.9 ± 10.0b
ensiling-NaOH	19.0 ± 0.3a,b	99.0 ± 0.9a,b	42.9 ± 0.2c	0.0c	17425.5 ± 40.3b	5428.1 ± 0.9b	471.8 ± 0.1a
	solid part after enzymatic hydrolysis					liquid part after enzymatic hydrolysis	
untreated	9.2 ± 0.1b	83.0 ± 0.0c	41.6 ± 0.2a	2.2 ± 0.1a,b	17002.0 ± 7.1c	16957.2 ± 35.0c	1860.3 ± 1.5a
ensiling	9.8 ± 0.1a	87.9 ± 0.4c	40.1 ± 0.0c	2.2 ± 0.0a,b	16465.0 ± 87.7c	10645.6 ± 18.7c	1431.6 ± 7.9c
NaOH	6.7 ± 0.0c	94.4 ± 0.2a	41.1 ± 0.0b,c	0.4 ± 0.0c	18370.0 ± 87.7a	40699.3 ± 222.7b	1527.9 ± 10.0c
ensiling-NaOH	6.5 ± 0.2c	93.0 ± 0.1b	41.3 ± 0.0b,c	0.4 ± 0.0c	17826.5 ± 24.8b	41222.1 ± 181.4a	1678.1 ± 7.5b
	solid part after ethanol fermentation and distillation					liquid part after ethanol fermentation and distillation	
untreated	8.9 ± 0.1c	77.7 ± 0.1c	41.0 ± 0.8a,b	2.1 ± 0.0c	16954.5 ± 17.7b	17145.3 ± 186.8c	1778.3 ± 7.2b
ensiling	10.6 ± 0.1a,b	82.6 ± 0.1c	40.5 ± 0.1a,b,c	2.0 ± 0.0c	17333.5 ± 98.3a	11056.6 ± 106.8c	1524.8 ± 13.8c
NaOH	10.3 ± 0.3a,b	89.1 ± 0.1a	39.5 ± 0.1c	2.3 ± 0.1a	16002.0 ± 80.2c	30989.9 ± 182.9b	1696.1 ± 5.8c
ensiling-NaOH	8.3 ± 0.3c	85.7 ± 0.4b	37.9 ± 0.5c	2.2 ± 0.0b	15647.0 ± 24.0c	33125.6 ± 62.8a	2040.0 ± 51.8a
	solid part after methane production					liquid part after methane production	
untreated		82.4 ± 0.1a	38.2 ± 0.0a,b	2.0 ± 0.0c	18509.5 ± 38.9c	1177.8 ± 0.9c	915.0 ± 6.1c
ensiling		81.6 ± 0.2b	38.6 ± 0.0a,b	2.0 ± 0.0c	19364.0 ± 52.3a	851.7 ± 4.8c	829.7 ± 7.6c
NaOH		77.3 ± 0.1c	37.6 ± 0.1c	2.2 ± 0.0a,b	18933.5 ± 57.3b	1536.8 ± 2.0a	1123.6 ± 10.7a
ensiling-NaOH		74.9 ± 0.1c	35.4 ± 0.2	2.1 ± 0.0a,bc	18298.5 ± 20.5c	1381.4 ± 14.6b	1043.3 ± 10.2b

a TS: total solid; VS: volatile solid; CV: calorific value; TC: total carbon; TN: total nitrogen. Letters a–d indicated that the values in the same column are significantly different (*p* < 0.05).

The TC concentration in the liquid fraction was 16 957.2 mg/L for the untreated sample and 10 645.6 mg/L for the ensiling treated sample after enzymatic hydrolysis (*p* < 0.05) and then increased to 17 145.3 and 11 056.6 mg/L after ethanol fermentation, respectively. After AD, the TC concentration significantly decreased to 1177.8 mg/L for the untreated sample and 851.7 mg/L for ensiling treated sample (*p* < 0.05). The TC concentration in the liquid fraction was 7240.0 mg/L for the NaOH treated sample and 5428.1 mg/L for the ensiling-NaOH treated sample and then increased to 30 989.9 and 33 125.6 mg/L after enzymatic hydrolysis and ethanol fermentation (*p* < 0.05). After AD, the TC concentration decreased to 1536.8 mg/L for NaOH treated sample and 1381.4 mg/L for ensiling-NaOH treated sample (*p* < 0.05). The TN concentration in the liquid part of *Pennisetum purpureum* was 1778.3 mg/L for the untreated sample and 1524.8 mg/L for ensiling treated sample after enzymatic hydrolysis and ethanol fermentation and then decreased to 915.0 and 829.7 mg/L after AD (*p* < 0.05), respectively. The TN concentration in the liquid fraction was 404.9 mg/L for NaOH treated sample and 471.8 mg/L for the ensiling-NaOH treated sample (*p* < 0.05) and then respectively increased to 1527.9 and 1678.1 mg/L after enzymatic hydrolysis. During the fermentation process, the TN concentration decreased from 1696.1 mg/L after ethanol fermentation to 1123.6 mg/L after AD for NaOH treated sample and from 2040.0 to 1043.3 mg/L for ensiling-NaOH treated sample (*p* < 0.05).

**Figure 4:**
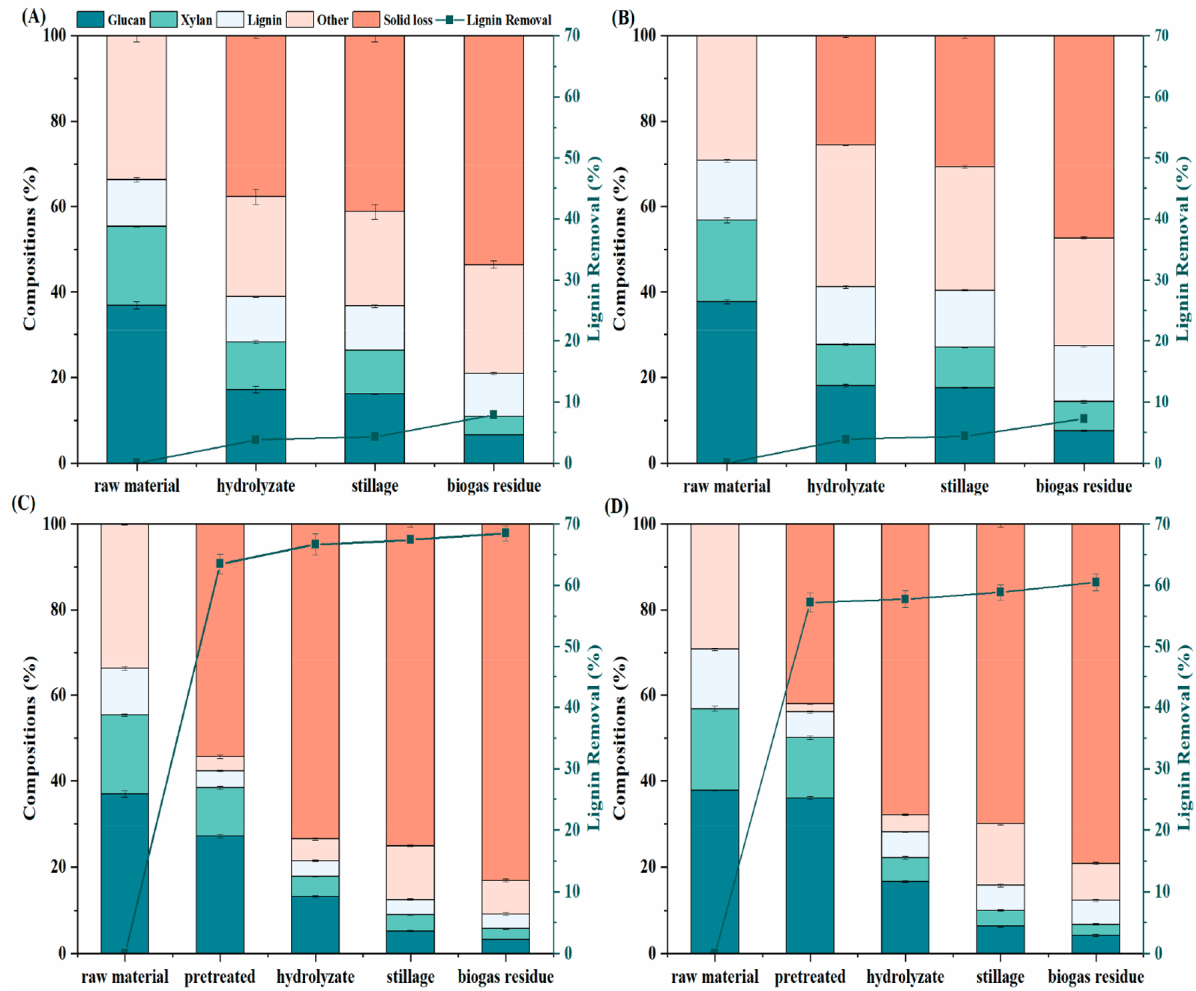
Compositions and lignin removal of *Pennisetum purpureum*. A: untreated sample; B: ensiling treated sample; C: NaOH treated sample; D: ensiling-NaOH treated sample.

As shown in Figure 4A and Table 3, the contents of glucan, xylan, and lignin in the untreated sample were 36.9%, 18.4%, and 10.9%. For the NaOH treated sample, the solid loss was 54.3%, of which the lignin removal was 63.5%. For the ensiling-NaOH treated sample, the solid loss was 42.0%, of which the lignin removal was 57.2%. This suggested that the mass loss in this stage is mainly due to the removal of lignin, which is consistent with the result reported by Scholl et al.^59^ During the enzymatic hydrolysis process, for ensiling treated sample, the glucan content sharply decreased from 37.7% to 18.1% (Figure 4B); for the NaOH treated sample, the glucan content dropped from 27.2% to 13.2%, and the glucan recovery was 73.6% (Figure 4C); for the ensiling-NaOH treated sample, the glucan content decreased from 36.0% to 16.7%, with a glucan recovery of 95.4% (Figure 4D). The significant decrease in glucan content (*p* < 0.05) could be explained by the hydrolysis of glucan into glucose (Figure 2). It should be pointed out that the lignin content was almost the same during enzymatic hydrolysis, ethanol fermentation, and the AD process (*p* > 0.05); this is because that lignin is poorly degradable in those processes.^60^

#### Material Flow under Different Production Processes

**Figure 5:**
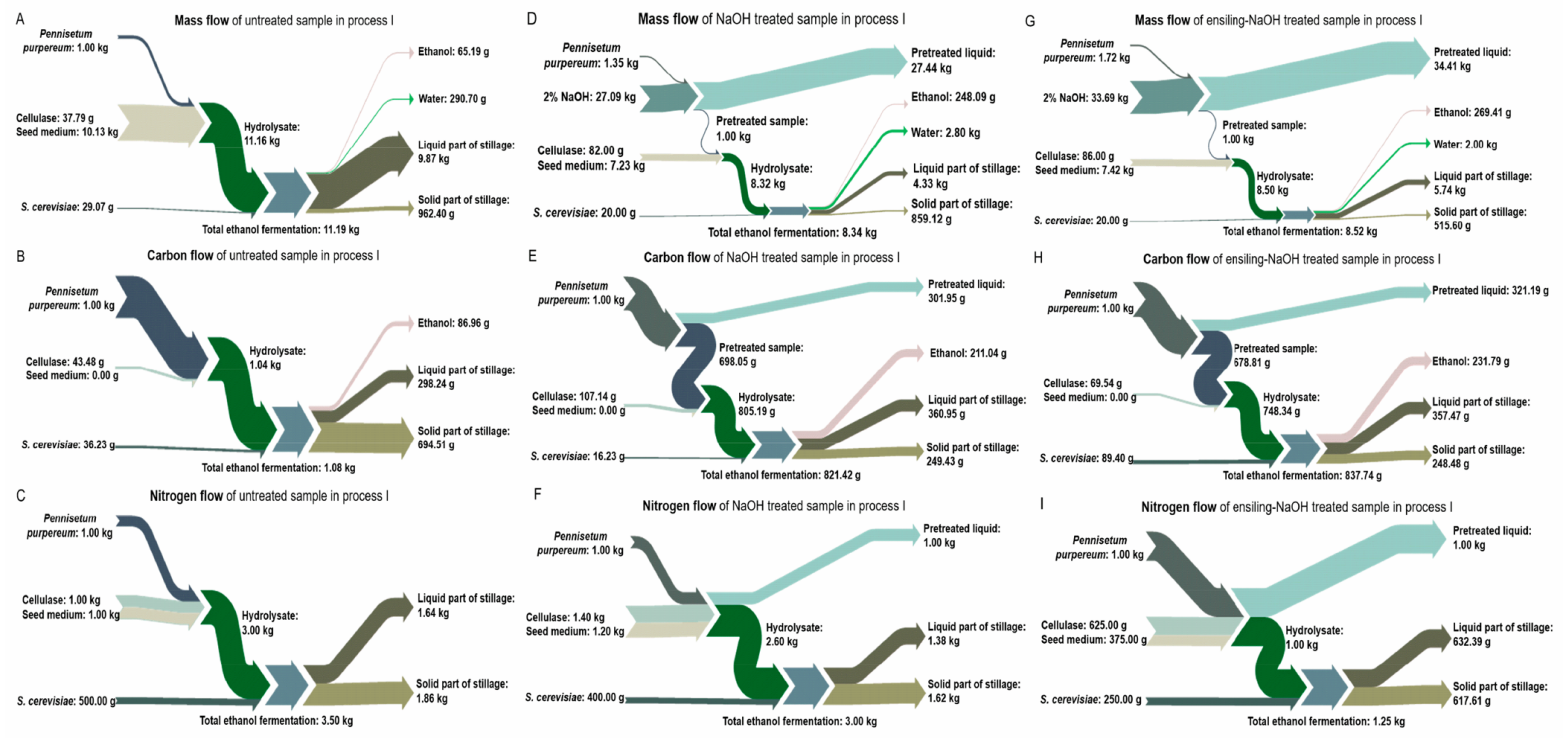
Mass (A, D, and G), carbon (B, E, and H), and nitrogen (C, F, and I) flow of untreated, NaOH treated, and ensiling-NaOH treated *Pennisetum purpureum* in ethanol production (process I). A–C represent the untreated sample; D–F represent the NaOH treated sample; G–I represent the ensiling-NaOH treated sample.

**Figure 6:**
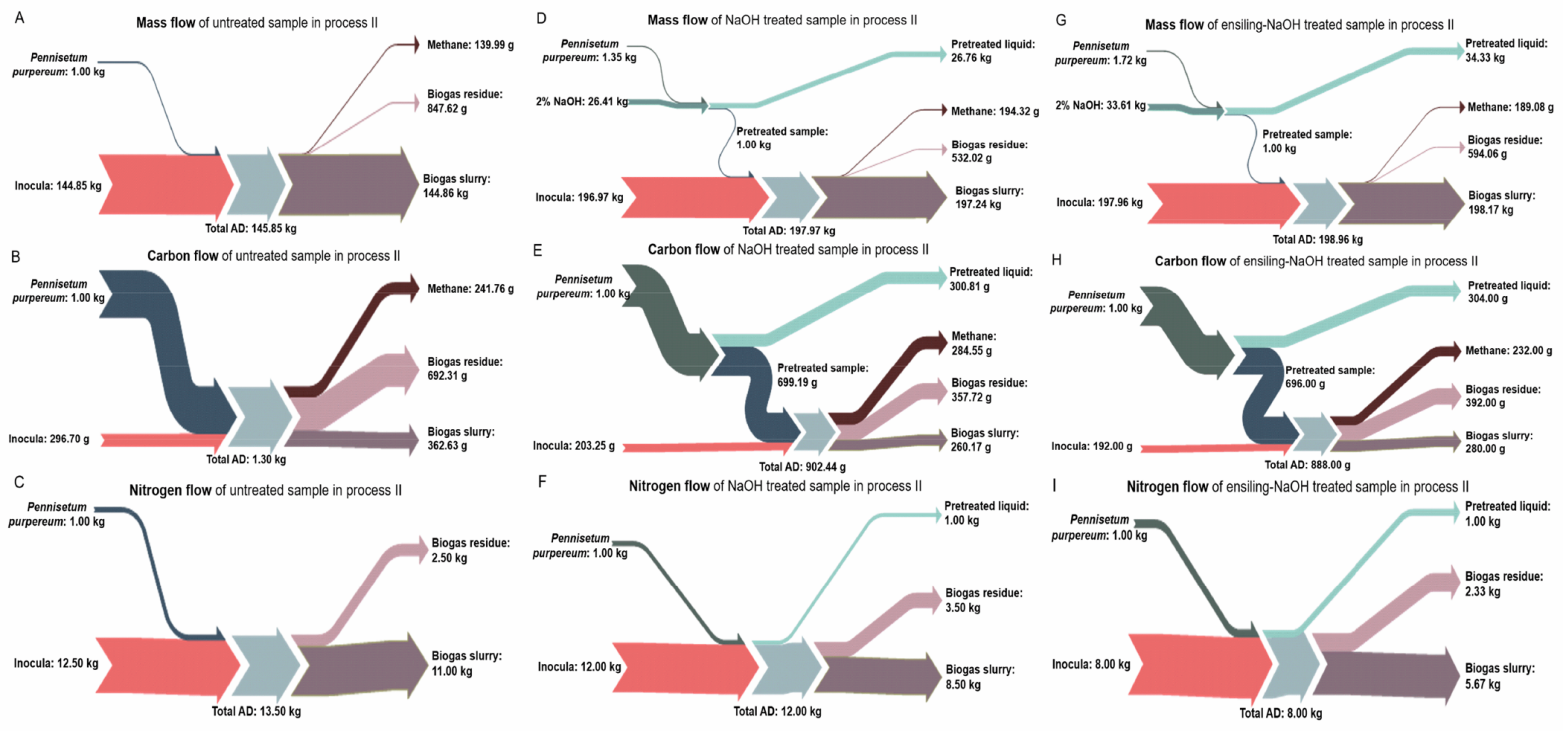
Mass (A, D, and G), carbon (B, E, and H), and nitrogen (C, F, and I) flow of untreated, NaOH treated and ensiling-NaOH treated *Pennisetum purpureum* in methane production (process II). A–C represent the untreated sample; D–F represent the NaOH treated sample; G–I represent the ensiling-NaOH treated sample.

**Figure 7:**
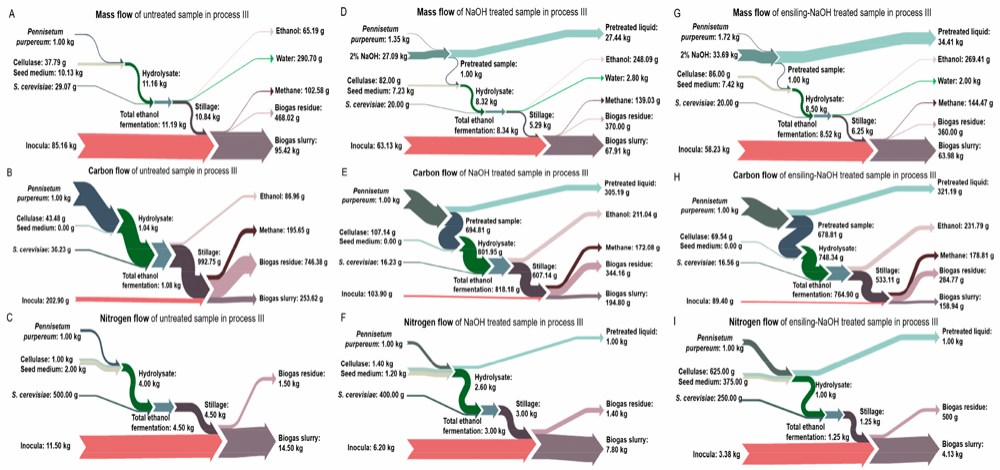
Mass (A, D, and G), carbon (B, E, and H), and nitrogen (C, F, and I) flow of untreated, NaOH treated and ensiling-NaOH treated *Pennisetum purpureum* in ethanol-methane coproduction (process III). A–C represent the untreated sample; D–F represent the NaOH treated sample; G–I represent the ensiling-NaOH treated sample.

Combining the content of TS, VS, C, N, TC and TN during the three processes, the mass flow including C and N flow could be achieved (Figures 5–7).

For process I, the flow of mass, carbon and nitrogen element are shown in Figure 5. After NaOH pretreatment (Figure 5D,G), the mass recovery was 45.7% for the untreated sample and 58.0% for ensiling treated sample; this further respectively decreased to 24.9% and 30.0% after enzymatic hydrolysis and ethanol fermentation. Considering the overall reaction system volume, it was observed that 65.2 g ethanol could be produced from 1 kg dry untreated sample, which increased to 248.1 g ethanol after NaOH pretreatment and 269.4 g ethanol after ensiling-NaOH pretreatment. For the carbon flow (Figure 5E,H), after NaOH pretreatment, 43.6% of carbon in the untreated sample and 56.7% of carbon in the ensiling treated sample remained in the solid residue, with 30.8% and 24.7% of carbon flowing into the liquid fraction, respectively; 13.2% of carbon in the untreated sample (accounting for 30.3% of the residue after NaOH pretreatment) and 18.6% of carbon in the ensiling treated sample (accounting for 32.8% of the residue after NaOH pretreatment) flowed into ethanol after enzymatic hydrolysis and fermentation. For the nitrogen flow (Figure 5F,I), all of the nitrogen in the untreated sample and ensiling treated sample flowed into the liquid fraction after NaOH pretreatment. Subsequently, all of the nitrogen flowed into the enzymatic hydrolysis process were from cellulase and seed medium.

The mass, carbon and nitrogen flow of process II are shown in Figure 6. After NaOH pretreatment (Figure 6D,G), the mass recovery was 45.7% for the untreated sample and 58.0% for the ensiling treated sample; this further respectively decreased to 28.1% and 34.6% after AD. Considering the overall reaction system volume, it was observed that 140.0 g of methane could be produced from 1 kg of dry untreated sample, which increased to 194.3 g methane after NaOH pretreatment and 189.1 g methane after ensiling-NaOH pretreatment. For the carbon flow (Figure 6E,H), after NaOH pretreatment, 14.9% of carbon in the untreated sample (accounting for 34.1% of the residue after NaOH pretreatment) and 18.7% of carbon in the ensiling treated sample (accounting for 33.1% of the residue after NaOH pretreatment) flowed into methane after AD. For the nitrogen flow (Figure 6F,I), all of the nitrogen in the untreated sample and ensiling treated sample flowed into the liquid fraction after NaOH pretreatment. Subsequently, all of the nitrogen flowing into the AD process was from the inoculum; after methane production process, 53.7% and 51.9% of the nitrogen in the inoculum flowed into the liquid fraction for the NaOH treated and ensiling-NaOH treated sample, respectively.

The mass, carbon and nitrogen flow of ensiling-NaOH treated *Pennisetum purpureum* samples under process III are shown in Figure 7. After NaOH pretreatment (Figure 7D,G), the mass recovery was 24.9% for the untreated sample and 30.0% for ensiling treated sample after enzymatic hydrolysis and ethanol fermentation; this further respectively decreased to 16.9% and 20.9% after AD. Considering the overall reaction system volume, it was observed that 65.2 g of ethanol + 102.6 g of methane could be produced from 1 kg of dry untreated sample, which increased to 248.1 g of ethanol + 139.0 g of methane after NaOH pretreatment and 269.4 g of ethanol + 144.5 g of methane after ensiling-NaOH pretreatment. This result was similar with the previous study conducted by Du et al.,^35^ who reported that 121.6 g of ethanol and 110.6 g of methane could be obtained from 1 kg of dried *Pennisetum purpereum*. For the carbon flow (Figure 7E,H), after NaOH pretreatment, 13.2% of carbon in the untreated sample (accounting for 30.3% of the residue after NaOH pretreatment) and 18.6% of carbon in the ensiling treated sample (accounting for 32.8% of the residue after NaOH pretreatment) flowed into ethanol after enzymatic hydrolysis and fermentation; meanwhile, 10.6% of carbon in the untreated sample (accounting for 24.4% of the residue after NaOH pretreatment) and 14.3% of carbon in the ensiling treated sample (accounting for 25.3% of the residue after NaOH pretreatment) flowed into methane after AD. For the nitrogen flow (Figure 7F,I), all of the nitrogen in the untreated sample and ensiling treated sample flowed into the liquid fraction after NaOH pretreatment. Subsequently, all of the nitrogen flowed into the enzymatic hydrolysis and fermentation process were from cellulase and seed medium, and the nitrogen during the AD process was from the inoculum.

#### Energy Flow under Different Production Processes

**Figure 8:**
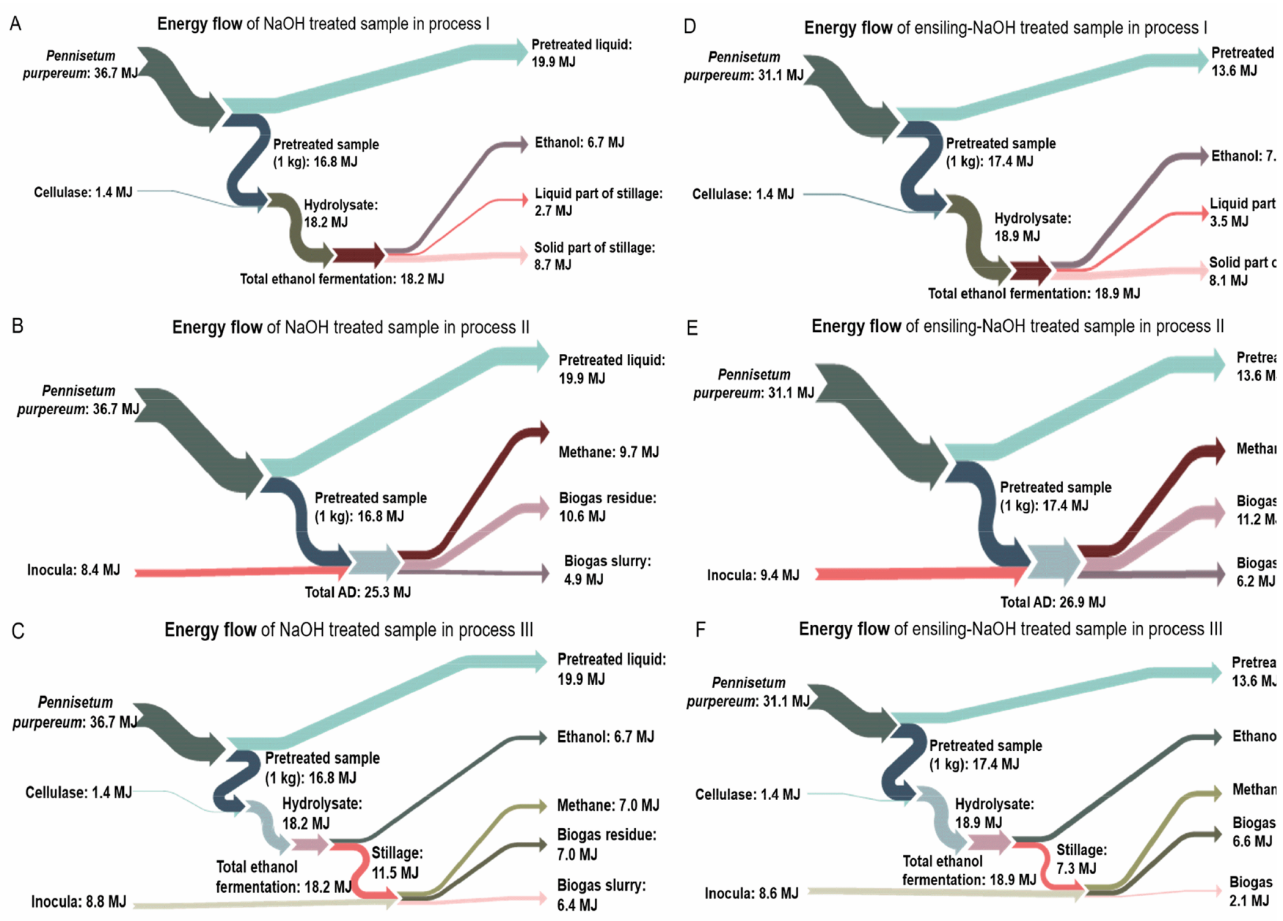
Energy flow of three processes of *Pennisetum purpureum*. A, D: process I; B, E: process II; C, F: process III. A–C represent the NaOH treated sample; D–F represent the ensiling-NaOH treated sample.

The energy flow under different production processes is shown in Figure 8. The energy values of ethanol and methane are 27.1 and 50.0 kJ/g, respectively.^61^ In this study, the energy value of raw *Pennisetum purpureum* is 16.8 MJ/kg, and only the energy output from ethanol and methane was calculated. As shown in Table 6, the energy production of 1.8 (10.5%), 7.0 (41.8%), and 6.9 (41.2%) MJ from 1 kg of untreated sample could achieve under processes I, II, and III; the energy production of 1.7 (9.5%), 6.9 (38.1%), and 6.7 (37.2%) MJ from 1 kg of ensiling treated sample could achieve under process I, II, and III; the energy produced from 1 kg of NaOH treated sample were 6.7 (39.9%), 9.7 (57.7%), and 13.7 (81.3%) MJ under three processes, and that from 1 kg of ensiling-NaOH treated sample was 7.3 (41.9%), 9.5 (54.3%), and 14.5 (83.4%) MJ under three processes, respectively. These results were similar to the literature when using other biomass for ethanol and methane production; for example, the energy yields of 5.1–5.2 and 8.8–9.3 MJ/kg were reported for oat straw and sugarcane bagasse.^36,62^ For the ensiling treated sample, the energy recovery from process III was 291.8% higher than those of process I, while the energies produced from processes II and III were similar. For NaOH treated sample, the energy production from process III increased by 103.4% compared with that from process I, and increased by 40.8% compared with that from process II. For the ensiling-NaOH treated sample, the energy production from process III increased by 98.9% compared with that from process I, and increased by 53.6% compared with that from process II. Comparing the three pretreatment methods and processes, the highest energy production was from the ensiling-NaOH treated sample in process III, which was 14.5 MJ/kg, with an energy conversion efficiency of up to 46.8% (accounting for 83.4% of residues after ensiling-NaOH pretreatment).

**Table 6:** Energy Output of Three Processes of *Pennisetum purpureum*

		energy output (energy conversion efficiency)^a^		
	pretreatment	process I/MJ	process II/MJ	process III/MJ
*Pennisetum purpureum*/kg	untreated	1.8 (10.5%)	7.0 (41.8%)	6.9 (41.2%)
	ensiling	1.7 (9.5%)	6.9 (38.1%)	6.7 (37.2%)
	NaOH	6.7 (39.9%)	9.7 (57.7%)	13.7 (81.3%)
	ensiling-NaOH	7.3 (41.9%)	9.5 (54.3%)	14.5 (83.4%)

a Energy conversion efficiency (%) = [ethanol and/or methane energy output]/[*Pennisetum purpurem* energy output] × 100%.

## Conclusions

Ethanol production, methane production, and coproduction of ethanol and methane were comparatively investigated from *Pennisetum purpureum* after ensiling, NaOH and ensiling-NaOH pretreatment. Results showed that there were no significant differences between NaOH pretreatment and ensiling-NaOH pretreatment in terms of the enhancement in ethanol production and methane production. However, the highest energy output was obtained via the coproduction of ethanol and methane process after ensiling-NaOH pretreatment; the production of ethanol and methane from 1 kg of ensiling-NaOH treated *Pennisetum purpureum* was 269.4 g of ethanol and 144.5 g of methane, respectively, which resulted in an energy output of 14.5 MJ with the energy conversion efficiency of 46.8%. These results demonstrated that the coproduction of ethanol and methane from *Pennisetum purpureum* outpaced the single ethanol and methane production, which may provide useful information for optimally exploiting its use for renewable biofuels production.

## Supporting Information Available

Figure S1: SEM (scanning electron microscope) images of untreated and pretreated *Pennisetum purpureum* (PDF)
