# Supplementary material for: Assessment of Coproduction of Ethanol and Methane from Pennisetum purpureum: Effects of Pretreatment, Process Performance, and Mass Balance
Source: ACS Sustain Chem Eng. 2021 Aug 5;9(32):10771–84. doi: 10.1021/acssuschemeng.1c02010 (PMC8815079; doi:10.1021/acssuschemeng.1c02010)
Supplement: Supplementary file 1 — sc1c02010_si_001.pdf [file sc1c02010_si_001.pdf]

Supporting Information for

Assessment of co-production of ethanol and methane from *Pennisetum purpureum*: effects of pretreatment, process performance and mass balance

Peiwen Wu [a,b,#], Xihui Kang [e,#*], Wen Wang [a,c,d], Gaixiu Yang [a,c,d], Linsong He [a,c,d], Yafeng Fan [a,c,d], Xingyu Cheng [a,c,d], Yongming Sun [a,c,d], Lianhua Li [a,c,d*]

[a] Guangzhou Institute of Energy Conversion, Chinese Academy of Sciences, No. 2, Nengyuan Road, Guangzhou 510640, China

[b] Key Laboratory of Ministry of Education for Water Quality Security and Protection in Pearl River Delta, Guangdong Provincial Key Laboratory of Radionuclides Pollution Control and Resources, School of Environmental Science and Engineering, Guangzhou University, No. 230, Wai Huan Xi Road, Guangzhou 510006, China

[c] Guangzhou Institute of Energy Conversion, CAS Key Laboratory of Renewable Energy, Chinese Academy of Sciences, No. 2, Nengyuan Road, Guangzhou 510640, P.R. China.

[d] Guangdong Key Laboratory of New and Renewable Energy Research and Development, No. 2, Nengyuan Road, Guangzhou 510640, P.R. China.

[e] MaREI Centre, Environmental Research Institute, University College Cork, 4 Lee Road, Sunday's Well, Cork, Ireland.

[#] The first two authors contributed equally to this paper

[*]Corresponding authors:

Dr. Xihui Kang: XKang@ucc.ie;

Dr. Lianhua Li, E-mail: lilh@ms.giec.ac.cn;

Number of pages: 2
Number of figures: 1

Surface structure Characterization of untreated and pretreated *Pennisetum purpureum*

Coated with gold in a high-resolution sputter coater (Cressington 108 Auto), the attached samples (both untreated and pretreated samples) were examined using a scanning electron microscope (JSM-6510, JEOL) operated at 5 kV.

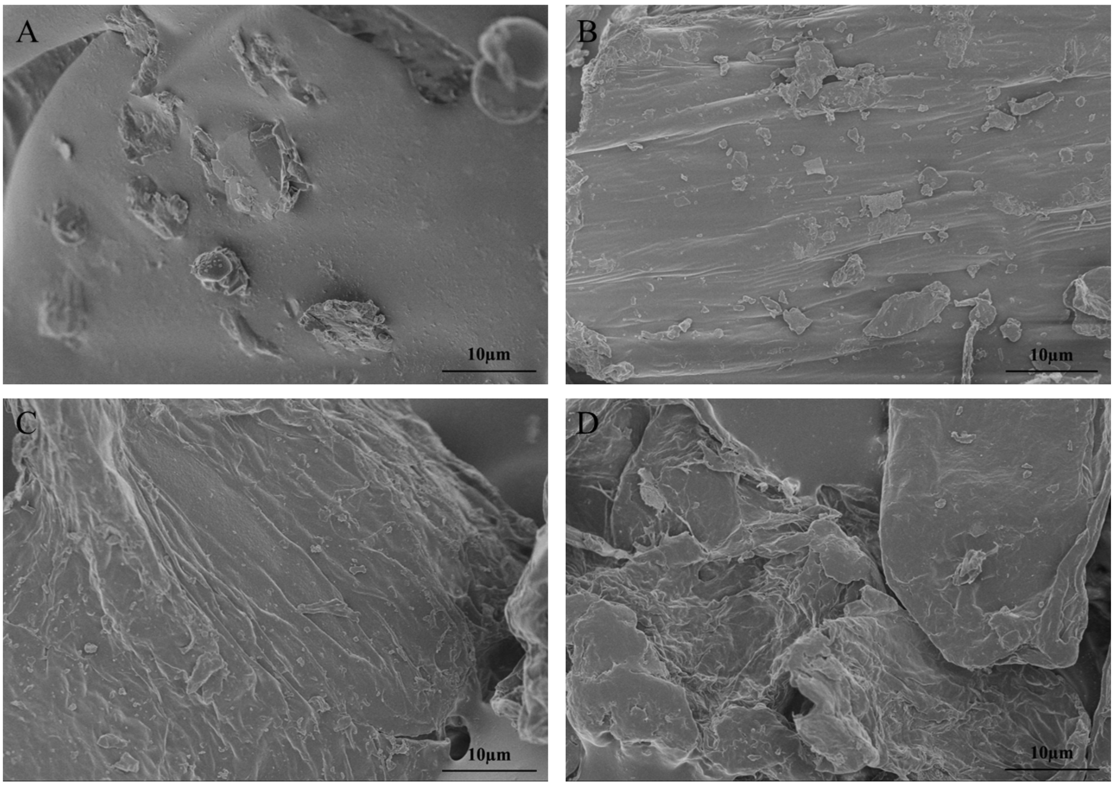

Figure S1. SEM (Scanning electron microscope) images of untreated and pretreated *Pennisetum purpureum*. A: untreated sample; B: ensiling treated sample; C: NaOH treated sample; D: ensiling-NaOH treated sample. Bar = 10 μm.

Figure S1 (A, B) shows that the surface structure of the untreated and ensiling treated sample is smooth and orderly, respectively. However, this surface structure became rough and irregular after NaOH pretreatment as shown in **Figure S1 (C, D)**.
